# Public Access to Summary Statistics for Genome-wide Association Studies of Body Mass Index, Weight, and Height Among Healthy Japanese Individuals: The Japanese Consortium of Genetic Epidemiology Studies

**DOI:** 10.2188/jea.JE20210459

**Published:** 2022-02-05

**Authors:** Atsushi Goto, Shiori Suzuki, Ryoko Katagiri, Taiki Yamaji, Norie Sawada, Masahiro Nakatochi, Kenji Wakai, Atsushi Hozawa, Kengo Kinoshita, Kozo Tanno, Atsushi Shimizu, Hidemi Ito, Keitaro Matsuo, Motoki Iwasaki

**Affiliations:** 1 Division of Epidemiology, National Cancer Center Institute for Cancer Control, Tokyo, Japan; 2 Department of Health Data Science, Graduate School of Data Science, Yokohama City University, Yokohama, Japan; 3 Division of Cohort Research, National Cancer Center Institute for Cancer Control, Tokyo, Japan; 4 Public Health Informatics Unit, Department of Integrated Health Sciences, Nagoya University Graduate School of Medicine, Nagoya, Japan; 5 Department of Preventive Medicine, Nagoya University Graduate School of Medicine, Nagoya, Japan; 6 Department of Preventive Medicine and Epidemiology, Tohoku Medical Megabank Organization, Tohoku University, Sendai, Japan; 7 Department of Integrative Genomics, Tohoku Medical Megabank Organization, Tohoku University, Sendai, Japan; 8 Division of Clinical Research and Epidemiology, Iwate Tohoku Medical Megabank Organization, Iwate Medical University, Iwate, Japan; 9 Division of Biomedical Information Analysis, Iwate Tohoku Medical Megabank Organization, Iwate Medical University, Iwate, Japan; 10 Division of Cancer Information and Control, Aichi Cancer Center Research Institute, Nagoya, Japan; 11 Division of Descriptive Cancer Epidemiology, Nagoya University Graduate School of Medicine, Nagoya, Japan; 12 Division of Cancer Epidemiology and Prevention, Aichi Cancer Center Research Institute, Nagoya, Japan; 13 Division of Cancer Epidemiology, Nagoya University Graduate School of Medicine, Nagoya, Japan

**Keywords:** body mass index, body weight, height, genome-wide association studies, Japanese

Recently, using the Mendelian randomization approach, we found that a genetically predicted high body mass index (BMI) was associated with an increased risk of colorectal cancer (CRC) in Japanese populations, strongly supporting the notion that high BMI influences the risk of CRC.^1^ In the study, we utilized data for single nucleotide polymorphism (SNP)-BMI associations from genome-wide association studies (GWAS) among 36,303 participants in the Japanese Consortium of Genetic Epidemiology studies (J-CGE). We also conducted GWAS in the J-CGE for SNP-height and SNP-weight associations. The J-CGE consists of data analyzed among healthy participants from the Tohoku Medical Megabank Community-Based Cohort (TMM) Study, the Japan Public Health Centre-based Prospective (JPHC) Study, the Japan Multi-Institutional Collaborative Cohort (J-MICC) Study, and the Hospital-based Epidemiologic Research Program at Aichi Cancer Center (HERPACC) Study (Table 1). In each study, BMI, body weight, and height were assessed at the baseline through health check-ups (83%) or self-administered questionnaires. Protocols for data harmonization, quality control of GWAS data, imputation, and statistical analysis were established in advance. For BMI, height, and body weight, after inverse normal conversion and without conversion (ie, crude values), a GWAS was conducted separately for each study and each sex, with age (continuous variable), age squared (continuous variable), and 10 to 20 principal components as covariates and anthropometric indices as outcomes, using multiple regression analysis. A meta-analysis was then conducted using a fixed-effects model for SNPs with the imputation quality above a certain level (INFO score ≥0.5).^2^ After quality control of the sample, the JPHC (3,525 men and 6,767 women), J-MICC (6,334 men and 7,736 women), TMM (3,123 men and 5,778 women), and HERPACC (1,502 men and 1,540 women) were combined to analyze 36,305 participants (Table 1). All the studies that contributed to J-CGE were approved by their respective institutional review boards.

**Table 1.** Characteristics of the studies utilized for the analyses

Study	BMI, kg/m^2^				Height, cm				Weight, kg			

	Men		Women		Men		Women		Men		Women	
	*N* (%)	Mean (SD)	*N* (%)	Mean (SD)	*N* (%)	Mean (SD)	*N* (%)	Mean (SD)	*N* (%)	Mean (SD)	*N* (%)	Mean (SD)
JPHC	3,524 (34.2)	23.6 (2.9)	6,766 (65.8)	23.8 (3.2)	3,525 (34.3)	162.1 (6.3)	6,766 (65.7)	150.6 (5.5)	3,525 (34.2)	62.3 (9.2)	6,767 (65.8)	54.1 (8.0)
J-MICC	6,334 (45.0)	23.8 (3.1)	7,736 (55.0)	22.5 (3.3)	6,334 (45.0)	167.4 (6.3)	7,736 (55.0)	154.8 (5.8)	6,334 (45.0)	66.8 (10.0)	7,736 (55.0)	53.8 (8.2)
TMM	3,123 (35.1)	24.1 (3.2)	5,778 (64.9)	23.2 (3.7)	3,123 (35.1)	165.1 (6.5)	5,778 (64.9)	153.1 (5.9)	3,123 (35.1)	65.9 (10.2)	5,778 (64.9)	54.4 (8.9)
HERPACC	1,502 (49.4)	23.5 (2.8)	1,540 (50.6)	22.0 (3.3)	1,502 (49.4)	167.6 (6.3)	1,540 (50.6)	156.1 (5.4)	1,502 (49.4)	66.2 (9.5)	1,540 (50.6)	53.5 (8.3)

BMI, body mass index; HERPACC, Hospital-based Epidemiologic Research Program at Aichi Cancer Center; J-MICC, Japan Multi-Institutional Collaborative Cohort Study; JPHC, Japan Public Health Centre-based Prospective Study; SD, standard deviation; TMM, Tohoku Medical Megabank Community-Based Cohort.

The results of the BMI and height GWAS were largely consistent with those reported by a previous large-scale Japanese GWAS among individuals with diseases.^3^^,^^4^ We have made the genome-wide summary data for SNP-BMI associations publicly available; SNP-height, and SNP-weight associations will also be made available.^5^ After excluding SNPs with minor allele frequency of <0.01, the number of SNPs included in the summary data were approximately 7,000,000. To the best of our knowledge, this is the first example of GWAS summary statistics made publicly available for BMI, height, and weight from a Japanese consortium comprised of a general population of Japanese individuals. The genome-wide summary data for BMI, weight, and height include coefficients, standard errors, and *P*-values, which are provided in the text files. The GWAS data among healthy Japanese individuals, such as those from the J-CGE Study, would be ideal for etiologic research with BMI, weight, or height as exposures or outcomes. These publicly available data can be used to perform genetic epidemiologic studies, such as Mendelian randomization studies, among Asian populations.
